# PhotonSABER: new tool shedding light on endocytosis and learning mechanisms *in vivo*

**DOI:** 10.1080/19420889.2019.1586048

**Published:** 2019-03-16

**Authors:** Shinji Matsuda, Wataru Kakegawa, Michisuke Yuzaki

**Affiliations:** a Department of Physiology, Keio University School of Medicine, Tokyo, Japan; b Department of Engineering Science, Graduate School of Informatics and Engineering, The University of Electro-Communications, Tokyo, Japan; c Brain Science Inspired Life Support Research Center (BLSC), The University of Electro-Communications, Tokyo, Japan

**Keywords:** AMPA receptor, Long-term depression, synapse, Purkinje cell, optogenetics

## Abstract

In the central nervous system, activity-dependent endocytosis of postsynaptic AMPA-type glutamate receptors (AMPA receptors) is thought to mediate long-term depression (LTD), which is a synaptic plasticity model in various neuronal circuits. However, whether and how AMPA receptor endocytosis and LTD at specific synapses are causally linked to learning and memory *in vivo* remains unclear. Recently, we developed a new optogenetic tool, PhotonSABER, which could control AMPA receptor endocytosis in temporal, spatial, and cell-type-specific manners at activated synapses. Using PhotonSABER, we found that AMPA receptor endocytosis and LTD at synapses between parallel fibers and Purkinje cells in the cerebellum mediate oculomotor learning. We also found that PhotonSABER could inhibit endocytosis of epidermal growth factor receptors in HeLa cells upon light stimulation. These results demonstrate that PhotonSABER is a powerful tool for analyzing the physiological functions of endocytosis in non-neuronal cells, as well as the roles of LTD in various brain regions.

## Text

Clathrin-mediated endocytosis is a cellular process in which cargo-containing clathrin-coated vesicles bud off from the plasma membrane and are transported to early endosomes [1]. This process occurs in all eukaryotic cells and plays essential roles in various cellular functions, such as intracellular signaling of growth factor receptors, uptake of nutrients, and membrane recycling. However, how endocytosis contributes to the functions of tissues and organs *in vivo* remains largely unclear because of the lack of tools that can acutely and reversibly regulate this process. In neurons, activity-dependent clathrin-mediated endocytosis of postsynaptic α-amino-3-hydroxy-5-methyl-4-isoxazolepropionic acid (AMPA)-type glutamate receptors (AMPA receptors) is thought to be the molecular basis of long-term depression (LTD) of synaptic transmission [2]. Although LTD occurs at synapses in various brain regions and is thought to serve as the cellular basis of learning and memory [3], whether and how LTD and AMPA receptor endocytosis at specific synapses are causally linked to learning and memory *in vivo* remains largely unclear. For example, LTD at synapses between parallel fibers and Purkinje cells is believed to mediate cerebellum-dependent oculomotor learning, such as the adaptation of optokinetic response (OKR) and vestibulo-ocular reflex (VOR) [4]. Many mutant mice in which cerebellar LTD is abrogated show impaired oculomotor learning [5,6]. Nevertheless, several lines of mutant mice that are defective in LTD in cerebellar slice preparations show normal oculomotor learning [7]. Discrepant LTD results may be caused by various LTD induction protocols used in *in vitro* slice preparations [8]. Alternatively, compensatory pathways at the molecular and circuitry levels may play a critical role in mice that have been genetically modified for their entire lives. Therefore, to clarify whether AMPA receptor endocytosis during LTD is directly linked to oculomotor learning, we developed a new tool that can acutely and reversibly control endocytosis at active synapses in a type-specific manner *in vivo*.

**Figure 1.**
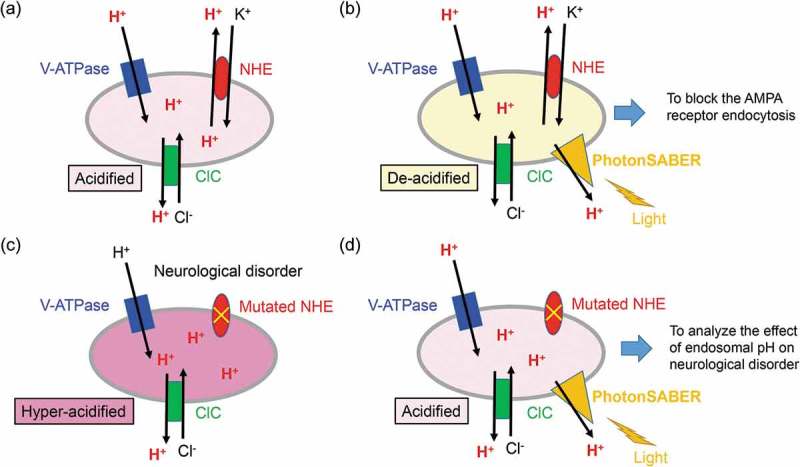
PhotonSABER de-acidifies the luminal pH of endosomes upon light stimulation. (a) PhotonSABER de-acidifies the luminal pH of endosomes upon light stimulation.The luminal pH is acidified by the coordinated activities of vacuolar-type H^+^-adenosine triphosphatase, chloride channels, and Na^+^/H^+^ exchangers (NHEs) in early to late endosomes in wild-type neurons. (b) The action of PhotonSABER in endosomes. The endosomal H^+^ is transported to the cytoplasm by light stimulation, and endosomal pH is elevated. Under this condition, endocytosis from the plasma membrane is severely impaired. (c) The function of NHEs is impaired in certain neurological disorders, leading to hyper-acidification of endosomes. (d) PhotonSABER is a useful tool for clarifying the pathogenesis of neurological disorders associated with defective NHEs. PhotonSABER is expected to rectify hyper-acidified endosomes and its associated phenotypes in neuronal cells derived from induced pluripotent stem cells from patients or from disease model mice.

The lumen of endosomes is normally maintained at acidic pH by using the cooperative functions of vacuolar vacuolar-type H^+^-adenosine triphosphatase (V-ATPase), chloride channels (ClCs), and Na^+^/H^+^ exchangers (NHEs) [9–11] (Figure 1(a)). Because the acidiﬁcation process is essential for endocytosis [12] and endosomal maturation [13], we hypothesized that endocytosis can be controlled by applying light stimulation to early endosomes that express light-driven H^+^ pumps. Thus, we developed a synthetic H^+^ pump, termed PhotonSABER, by fusing an engineered Anabaena sensory rhodopsin (ASR) in which Glu has replaced Asp217 (ASR^D217E^) to direct H^+^ flow from the extracellular to the cytoplasmic direction, [14] and the C-terminal intracellular domain of ClC5 carries the endosome localization signal [15], The light stimulation of hippocampal neurons or cerebellar Purkinje cells expressing PhotonSABER elevated the pH in endosomes and inhibited activity-dependent AMPA receptor endocytosis (Figure 1(b)). Furthermore, fiberoptic illumination of the cerebellar flocculus of knock-in mice, in which PhotonSABER was specifically expressed in Purkinje cells, inhibited oculomotor learning during OKR and VOR adaptation. Whereas the number of postsynaptic AMPA receptors decreased in the flocculus after OKR adaptation in wild-type mice, it was unchanged in light-stimulated PhotonSABER knock-in mice. These results clearly indicated that AMPA receptor endocytosis during LTD, which was inhibited by light-stimulated PhotonSABER, is directly responsible for oculomotor learning *in vivo*.

PhotonSABER can be used to assess the physiological importance of LTD in various brain regions. PhotonSABER can be expressed in specific cells by mating PhotonSABER knock-in mice, which contain the stop sequence flanked by loxP sites, with brain-region- and cell-type-specific *Cre* driver mice. Light stimulation acutely and reversibly inhibits LTD induction without affecting the normal development of neuronal circuits. For example, LTD at synapses between Schaffer collaterals and CA1 neurons in the hippocampus is proposed to be required for consolidation of context-dependent fear memory, but not for its acquisition [16]. LTD at these synapses is also reported to be required for consolidation of spatial memory [17]. Knock-in mice that express PhotonSABER in CA1 neurons are useful for directly examining whether and exactly when AMPA receptor endocytosis mediates these hippocampus-dependent memories *in vivo*. The amygdala is believed to be responsible for cue-dependent fear memory. Stimulation of auditory input to the amygdala without foot shock has been shown to induce LTD at these synapses to facilitate the extinction of its associated fear memory [18]. PhotonSABER knock-in mice are also useful for clarifying whether and exactly when AMPA receptor endocytosis mediates the extinction of fear memory *in vivo*.

Notably, the mutation of NHEs is reported to be linked with human neurological and neuropsychiatric disorders. For example, a mutation in the *NHE6* gene has been identified in Angelman syndrome patients [19]. Mutations in *NHE7* and *NHE9* are reported to be linked with Alzheimer’s disease and autism spectrum disorders, respectively [20,21]. A major function of NHEs is to pump out H^+^ to the cytoplasm and pump in K^+^ into the endosomal lumen. Thus, the endosomal lumen of patients with these diseases may be hyper-acidified (Figure 1(c)). By expressing PhotonSABER in neurons differentiated from patient-derived induced pluripotent stem cells or disease model mice, we could address whether and how the endosomal pH is related to disease phenotypes (Figure 1(d)). Therefore, PhotonSABER is a powerful tool for clarifying the pathogenesis of certain neurological and neuropsychiatric disorders and developing new therapeutic targets.

**Figure 2.**
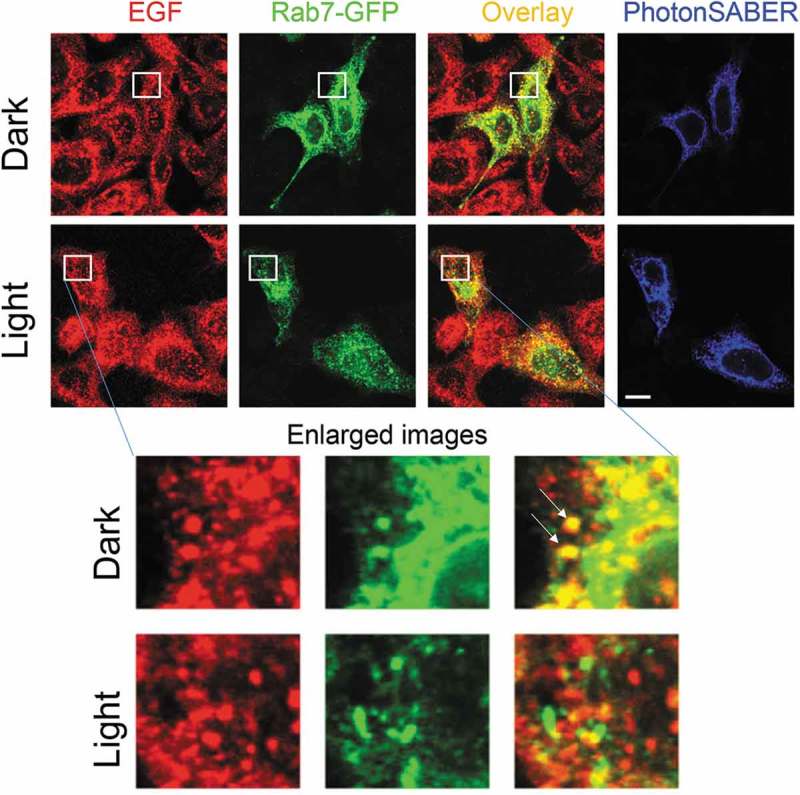
Light stimulation inhibits epidermal growth factor (EGF) endocytosis in HeLa cells expressing PhotonSABER. Light stimulation reduces colocalization of EGF and Rab7-green fluorescent protein (Rab7-GFP) in HeLa cells. HeLa cells expressing hemagglutinin-tagged PhotonSABER and Rab7-GFP were incubated with biotinylated EGF for 60 min. The regions marked by white squares are magnified in the bottom panels. Arrows indicate the colocalization of Rab7-GFP with EGF. The bar represents 10 μm.

The application of PhotonSABER is not limited to neurons. For example, phagocytosis of dextran can be inhibited by light stimulation of various cell lines expressing PhotonSABER (data not shown). Many growth factors are taken up by cells via endocytosis of growth-factor-bound cell-surface receptors. When HeLa cells were incubated with biotinylated recombinant epidermal growth factor (EGF) for 60 min, EGF signals were detected in Rab7-positive late endosomes. However, when light was applied to HeLa cells expressing PhotonSABER, most EGF signals were not internalized and the internalized signals were not colocalized with Rab7 (Figure 2). Although two pathways for EGF signaling exist, one mediated by cell-surface EGF receptors and another by those in endosomes, how these pathways differentially contribute to the EGF signaling cascade remains unclear [22]. Therefore, PhotonSABER is a useful tool for clarifying the contribution of signaling endosomes.

Why and how does the lumen of early endosomes need to be acidified for their functional maturation? Adenosine diphosphate ribosylation factor nucleotide binding site opener, a guanine exchange factor for the small G proteins of the adenosine diphosphate ribosylation factor family [23], is reported to be recruited to acidified endosomes via its interaction with V-ATPase [24,25], but the precise underlying mechanisms remain unclear. PhotonSABER is also a useful tool for addressing these fundamental cell biology questions. Light-induced synchronization of pH of endosomes enables easier proteomic analysis of acidification-related endosomal proteins. Furthermore, live imaging of cells expressing PhotonSABER enables the visualization of pH-dependent recruitment or exclusion of endosomal proteins.

Additional studies are warranted to expand the concept of optogenetic pH regulation of early endosomes to other types of endosomes, such as recycling and late endosomes, to modulate exocytosis and lysosomal trafficking, respectively, and clarify their physiological functions *in vivo*.

## Materials and methods

Regarding endocytosis of EGF, HeLa cells expressing hemagglutinin (HA)-tagged PhotonSABER and Rab7-green fluorescent protein were treated with biotinylated EGF (2 μg/mL; Molecular Probes, Eugene, OR, USA) for 60 min in the presence or absence of light stimulation (2000 lx). Cells were fixed with 4% paraformaldehyde in phosphate buffered saline (PBS) for 10 min at room temperature. After being washed with PBS three times, cells were blocked with PBS containing 2% bovine serum albumin, 2% normal goat serum, and 0.4% Triton-X100. To label HA-tagged PhotonSABER, cells were incubated with rat monoclonal anti-HA antibodies (1:1,000; Roche, Basel, Switzerland) for 60 min and visualized with Alexa405-conjugated secondary antibodies (1:1,000; Molecular Probes). Biotinylated EGF was visualized using Alexa554-conjugated streptavidin (1:1,000; Molecular Probes). In the representative images, constant levels of brightness and contrast were used throughout each experimental series.
